# Widespread Dominance of Kinetoplastids and Unexpected Presence of Diplonemids in Deep Freshwater Lakes

**DOI:** 10.3389/fmicb.2019.02375

**Published:** 2019-10-16

**Authors:** Indranil Mukherjee, Yoshikuni Hodoki, Yusuke Okazaki, Shohei Fujinaga, Kako Ohbayashi, Shin-ichi Nakano

**Affiliations:** ^1^Center for Ecological Research, Kyoto University, Otsu, Japan; ^2^Bioproduction Research Institute, National Institute of Advanced Industrial Science and Technology, Tsukuba, Japan; ^3^Department of General Systems Studies, The University of Tokyo, Tokyo, Japan

**Keywords:** 18S amplicon sequencing, CARD-FISH, deep lakes, diplonemids, flagellates, hypolimnion, kinetoplastids

## Abstract

Kinetoplastid flagellates are generally abundant in the deep sea and recently they were even found to be dominant in the hypolimnion of a deep freshwater lake. Therefore, to understand the distribution of kinetoplastids in deep freshwater lakes, we have collected vertical samples from five lakes in Japan. The abundance of kinetoplastids was enumerated by Catalyzed Reporter Deposition-Fluorescence *in situ* Hybridization, and the diversity was determined by 18S amplicon sequencing using universal eukaryote and kinetoplastid-specific primers. Kinetoplastids were abundant in the deep waters of all the lakes, contributing up to 53.6% of total nanoeukaryotes. Despite this significant contribution, kinetoplastids remain undetected by amplicon sequencing using universal primers that are widely used in eukaryotic diversity studies. However, they were detected with specific primers, and the communities were characterized by both ubiquitous and lake-specific unique OTUs. Oligotyping of a ubiquitous and dominant OTU revealed the presence of lake-specific sequence types (oligotypes). Remarkably, we also detected diplonemids (a sister group of kinetoplastids and considered to be specific in the marine habitat) using kinetoplastid-specific primers, showing their presence in freshwaters. Underestimation of kinetoplastids and diplonemids using universal primers indicates that euglenozoan flagellates are overlooked in diversity studies worldwide. The present study highlighted the importance of kinetoplastids in the hypolimnion of deep lakes, thereby indicating their role in material cycling in deep waters.

## Introduction

Diversity studies in microbial eukaryotes conducted recently by various molecular techniques have shown immense diversity of protists (Moon-van der Staay et al., 2001; Moreira and Lopez-Garcia, 2002; Grossmann et al., 2016) and identified several important lineages. Several of these recently reported lineages were previously unknown when the studies were mainly culture-based and employed light microscopy for identification (Massana et al., 2006; de Vargas et al., 2015). However, the universal eukaryote primers commonly used to understand the diversity of these organisms often underestimate several lineages owing to the divergent nature of their 18S rRNA gene (Berney et al., 2004; Simpson et al., 2006; Bochdanksy and Huang, 2010). This might not create a serious problem if minor groups are underestimated, but can display a different picture of the ecosystem processes if abundant groups are ignored.

One particular flagellate group, kinetoplastids, have regularly been undetected in nearly all the diversity studies in microbial eukaryotes using molecular techniques (Von der Heyden and Cavalier-Smith, 2005; Vaerewijck et al., 2008; Mukherjee et al., 2015). Kinetoplastids are among the most cosmopolitan flagellates in aquatic environments (Patterson and Larsen, 1991; Arndt et al., 2000) with high diversity (Von der Heyden and Cavalier-Smith, 2005). An important aspect of kinetoplastid phylogeny is the massive evolutionary change of their 18S rRNA gene, due to which they are found at the base of the eukaryotic phylogenetic trees (Simpson et al., 2006; Janouškovec et al., 2017; Strassert et al., 2019). Kinetoplastids, along with diplonemids and euglenids belong to the phylum Euglenozoa (Cavalier-Smith, 1981), where the free-living and occasionally parasitic group diplonemids are their closest relatives (Lara et al., 2009). The euglenozoans diverged from other eukaryotes (Moreira et al., 2004; Vlcek et al., 2010) and are known as basal eukaryotes (Cavalier-Smith, 2009).

Class Kinetoplastea consists of parasitic, uniflagellate trypanosomatids and free-living, biflagellate bodonids (Vickerman, 1976). Trypanosomatids are well studied owing to their medical importance, whereas bodonids have received limited attention (Simpson et al., 2006). Bodonids are heterotrophic and are often found to dominate in cultures. Owing to their opportunistic nature, several stains have been cultured and a few laboratory studies have been conducted to understand their ecology (Caron, 1987; Boenigk and Arndt, 2000; Zubkov and Sleigh, 2000). Although these flagellates are cosmopolitan with high diversity they are considered to have lower abundance in the aquatic ecosystems (Arndt et al., 2000). Recent studies reported that owing to the mismatches in their 18S rRNA gene the commonly used universal eukaryote primers and probes do not target kinetoplastids (Bochdanksy and Huang, 2010; Mukherjee et al., 2015). This has led to their underestimation in diversity and abundance-based studies worldwide and is likely the reason for there being fewer reports on them in natural environments in such studies. This group has recently attracted some interest among researchers as using specific primers and Catalyzed Reporter Deposition-Fluorescence *In Situ* Hybridization (CARD-FISH) probes kinetoplastids were reported to have high abundance and diversity in the deep sea (López-García et al., 2003; Edgcomb et al., 2011; Morgan-Smith et al., 2011, 2013; Salani et al., 2012) and also from the hypolimnion of a freshwater deep holomictic lake (Mukherjee et al., 2015). The results from these studies suggest that kinetoplastid flagellates mainly inhabit the deep waters of both oceans and lakes. However, limited studies from the deep waters of freshwater lakes have restricted our understanding of the hypolimnion kinetoplastid communities, therefore indicating the need for further studies to understand the importance of these less-studied flagellates, especially from the deeper waters of freshwater lakes.

Owing to the water mixing patterns in freshwater deep holomictic lakes, where water column is mixed in some seasons and remain stratified in other seasons, the microbial communities in different depths undergo community assembly and re-assembly. This seasonal community variation in different depths presents an interesting ecosystem to understand the ecology of these microorganisms. However there are only a few studies on freshwater deep lakes, and the microbial communities in the hypolimnion waters are poorly understood (Okazaki and Nakano, 2016; Mukherjee et al., 2017). The limited number of studies that have been conducted on the oxygenated hypolimnion of freshwater deep lakes have studied bacterial community, where they reported the dominance of hypolimnion-specific bacterial lineages (Urbach et al., 2001; Callieri et al., 2015; Okazaki et al., 2017; Mehrshad et al., 2018; Andrei et al., 2019). Information about the protist communities in hypolimnion waters is poorly known. However, the limited information available about the protist communities in the oxygenated hypolimnion waters of some deep holomictic lakes also indicates the dominance of hypolimnion-specific lineages (Lepère et al., 2010; Mukherjee et al., 2015, 2017).

**FIGURE 1:**
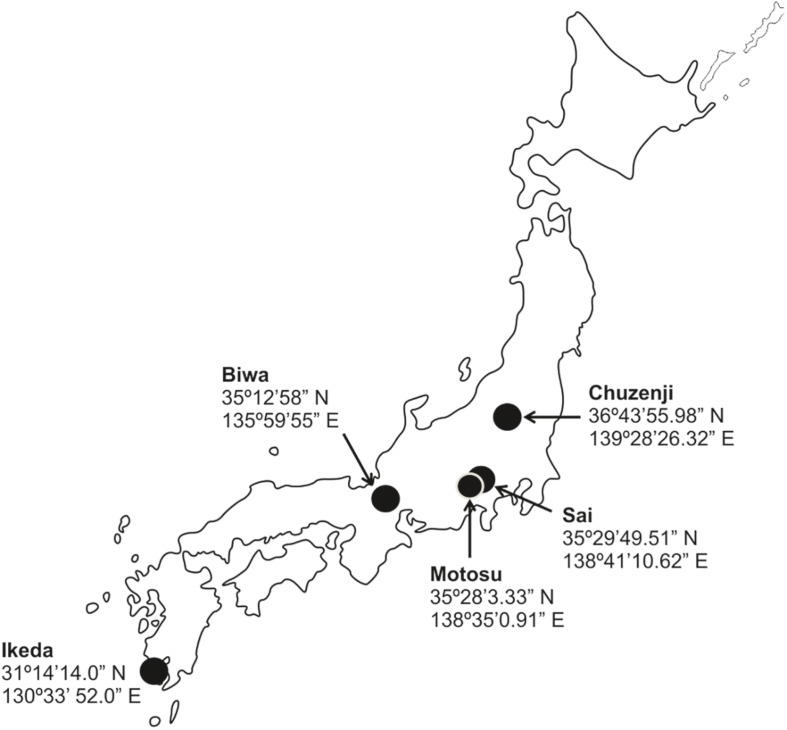
Map of Japan with the location of the sampled lakes.

To understand the abundance and distribution of kinetoplastids in deep freshwater lakes, we took water samples of various depths from five deep freshwater lakes in Japan (Figure 1) with oxygenated hypolimnion during summer stratification (Table 1). The abundance of kinetoplastids was examined using group-specific CARD-FISH probes and their detailed community composition was analyzed by 18S rRNA gene amplicon sequencing with universal eukaryote and kinetoplastid-specific primers. Moreover, oligotyping was conducted on ubiquitous kinetoplastid OTU to understand whether geographically isolated deep waters of freshwater lakes exhibit sequence variation among the OTUs.

**TABLE 1:** Characteristics and sampling information of each lake.

**Lake**	**Trophic status**	**Surface area (km^2^)**	**Z max (m)**	**Sampling month and year**	**Mean bacterial abundance (× 10^6^ cells ml^−1^)**	**Mean conc. (till 30 m) of chlorophyll *a* (μg l^–1^)**
Biwa	Mesotrophic	674.0	104	August and November 2014	2.0 ± 1.3	0.8 ± 0.5
Chuzenji	Oligotrophic	11.62	161	August 2014	1.3 ± 0.6	0.8 ± 0.4
Motosu	Oligotrophic	4.7	121	October 2014	0.7 ± 0.1	0.5 ± 0.9
Sai	Oligotrophic	2.1	74	October 2014	1.4 ± 0.4	1.1 ± 0.7
Ikeda	Mesotrophic	11.0	200	July 2015	2.8 ± 3.2	1.9 ± 1.5

## Materials and Methods

### Study Site and Sampling

**FIGURE 2:**
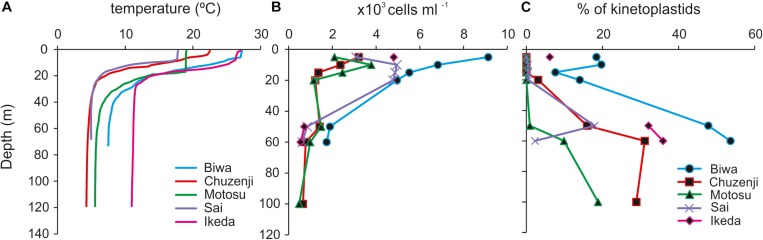
Vertical profile of **(A)** temperature; **(B)** abundance of nanoeukaryotes; **(C)** percentage contribution of kinetoplastids to total nanoeukaryotes in the studied lakes. Sampling depths are indicated by symbols. Sampling depths of each lake covering the epilimnion and hypolimnion are: Biwa (5, 10, 15, 20, 50, and 60 m), Chuzenji (5, 10, 15, 20, 50, 60, and 100 m), Motosu (5, 10, 15, 20, 50, 60, and 100 m), Sai (5, 10, 20, 50, and 60 m), and Ikeda (5, 50 and 60 m).

Samples were collected from five monomictic deep freshwater lakes in Japan with oxygenated hypolimnion (Figure 1 and Table 1). Samples from various depths covering the epilimnion and hypolimnion were taken once from one station in each lake (except for Lake Biwa where sampling was conducted twice) during the thermal stratification period (Figure 2A and Table 1). Sampling in Lake Biwa was conducted from station Ie-1 (a long-term limnological survey station of Kyoto University, Japan) and from a station located at the deepest part for all the other lakes. Samples were collected with a 5 liter Niskin sampler (General Oceanics, Miami, United States) and the hydrographic structure was determined with a conductivity-temperature-depth profiler (911 plus, Sea Bird Electronics, Inc., United States) for Lake Biwa and with a Rinko profiler (ASTD 102, JFE Advantech Co., Ltd., Japan) for the other lakes. The concentration of chlorophyll *a* was measured simultaneously using the CTD and Rinko profiler (Supplementary Figure S1A and Table 1). Samples were collected in clean plastic bottles, which were rinsed three times with sample water before collection and were kept cool and dark in an icebox and transported to the laboratory within a few hours of collection.

### Total Bacteria Count

Samples were fixed immediately after collection with glutaraldehyde (1% final concentration) and stored at 4°C until filtration. A 1 ml water sample was filtered through a polycarbonate membrane filter (pore size 0.2 μm, diameter 25 mm, Advantec), and stained with 4, 6-diamidino-2-phenylindole (DAPI) (Porter and Feig, 1980). The bacterial cells were observed under UV light with an epi-fluorescent microscope (Olympus BX- 50, Japan). Duplicate counts from each sample were counted at 1000 × magnification from 20 randomly chosen fields (a minimum of 300 cells were counted) (Supplementary Figure S1B and Table 1).

### DNA Extraction and Pyrosequencing

In Lake Biwa, owing to the relatively high abundance of kinetoplastids in the hypolimnion from August and November and also in the epilimnion from August (Mukherjee et al., 2015), DNA samples were collected from both the epilimnion and hypolimnion in August and only from the hypolimnion in November (Table 2). DNA samples from the four other lakes were collected from one depth in the hypolimnion (Table 2). Water samples were pre-filtered just after collection using a 20-μm mesh plankton net to exclude bigger organisms. One to two liters (500 ml for Lake Ikeda) of water samples were filtered with a 0.8 μm polycarbonate filter (47 mm diameter, Costar) at low vacuum (7.5 cm Hg) and immediately frozen at −30°C until analysis. DNA was extracted using a Power Soil DNA isolation and purification kit (MoBio laboratories, Carlsbad, CA, United States) and quantified using a Nanodrop ND-1000 spectrophotometer (NanoDrop Technologies, Inc., Wilmington, DE, United States). Separate polymerase chain reactions (PCR) were conducted for general eukaryotes and kinetoplastids. PCR was conducted using universal eukaryote primers TAReuk454FWD1 (5′-CCAGCA(G/C)C(C/T)GCGGTAATTCC-3′) and TAReukREV3 (5′-ACTTTCGTTCTTGAT(C/T)(A/G)A-3′) to amplify the V4 region of the 18S rRNA gene of all eukaryotes (Stoeck et al., 2010). Samples from Lake Ikeda were not used for the analysis of the total eukaryotic community. The kinetoplastid 18S rRNA gene was amplified using kinetoplastid-specific primers, kineto14F (Von der Heyden and Cavalier-Smith, 2005) and Kin500R (Scheckenbach et al., 2005) to amplify the V1 and V2 variable regions. Adapters for the 454 pyrosequencer were attached to both the forward and reverse primers and sample-specific barcode tags were attached only to the forward primers. PCRs were performed in 25 μl of reaction volume with a Blend Taq PCR kit (Toyobo, Osaka, Japan). The PCR conditions for the universal eukaryote primers were: initial denaturation at 98°C for 30 s, 10 cycles (98°C for 10 s, 53°C for 30 s, and 72°C for 2 s), then 15 similar cycles with a 48°C annealing temperature, and a final extension at 72°C for 10 min (Massana et al., 2015). The PCR conditions for the Kinetoplastid-specific primers were 94°C for 5 min, 35 cycles (94°C for 30 s, 69°C for 36 s, and 72°C for 4.5 min), and a final extension at 72°C for 10 min (Von der Heyden and Cavalier-Smith, 2005). For the Lake Ikeda samples, owing to the lower concentration of kinetoplastid template DNA, the number of PCR cycles was increased to 40. Amplicons were checked using agarose gel electrophoresis and DNA was purified using an UltraClean PCR Clean-Up kit (MoBio Laboratories, Carlsbad, CA, United States). Samples were quantified using a Nanodrop ND-1000 spectrophotometer (NanoDrop Technologies, Inc., Wilmington, DE, United States). The samples were pooled to have uniform DNA concentration in each sample and were sent to Macrogen, Japan for pyrosequencing using a Roche 454 GS-FLX titanium system (Macrogen Japan Corp. Kyoto, Japan). The sequence data were submitted to the DDBJ Sequence Read Archive (DRA) database and are available under the Bio-project accession number: PRJDB6819.

**TABLE 2:** Details of individual libraries with kinetoplastid-specific primers.

**Sample**	**Depth (m)**	**No. of OTUs**	**Unique OTUs**	**Euglenozoan sequences**	**Shannon diversity**	**Richness S.chao1**
Biwa Epi	5	6	1	458	2.07	17
Biwa Aug Hypo	60	15	3	1558	1.09	24.33
Biwa Nov Hypo	60	19	10	1036	0.95	25.33
Chuzenji	70	5	1	2555	0.78	4.0
Motosu	100	1	0	181	0.08	3.0
Sai	65	4	0	293	0.63	5.0
Ikeda	50	8	2	286	1.72	9.0

*Biwa Epi, Lake Biwa epilimnion; Biwa Aug Hypo, Lake Biwa August hypolimnion; Biwa Nov Hypo, Lake Biwa November hypolimnion.*

### Pyrotag Processing

The processing and quality control of the sequencing data were conducted using UPARSE (Edgar, 2013). Sequences were de-multiplexed using the bar code identifier in the forward primer. A chimera check was conducted with UCHIME (Edgar et al., 2011) using *de novo* and reference-based chimera searches against the PR2 database (Guillou et al., 2013). Pyrotags were trimmed to have 300 bp of a good quality sequence, and pyrotags with less than 200 bp lengths were excluded from further analysis. OTUs were then clustered with 97% similarity with USEARCH (Edgar, 2010). Classification of each OTU was conducted against the SILVA reference database^1^. The unknown OTUs were separately classified to their closest relatives with BLAST searches against the NCBI nucleotide collection (nt) database^2^.

### Oligotyping

Oligotyping was conducted to understand the intra-diversity within a particular OTU by analyzing the single nucleotide variation, excluding the sequencing errors based on Shannon entropy values (Eren et al., 2013). The dominant kinetoplastid OTU was selected for oligotyping following the author’s instructions^3^. The quality filtered FASTA files of the individual reads in each OTU generated by the UPARSE pipeline were aligned using online aligner SINA 1.2.11 (Pruesse et al., 2012). Gaps in the aligned sequences were eliminated and sequences were trimmed using the scripts “o-trim-uninformative-columns-from-alignment” and “o-smart-trim” scripts, respectively. The most informative column with the highest entropy value was chosen from several rounds of oligotyping until all oligotypes with >100 reads exceeded the purity score of 0.90.

### Phylogenetic Analysis

Phylogenetic analysis was conducted with 18S rRNA gene sequence of diplonemids closely related to the diplonemid OTU obtained in the present study and also with the sequences of cultured diplonemids. Kinetoplastids and other euglenozoan sequences were considered as an outgroup. Sequences were aligned using MAFFT (LINSI) (Katoh and Toh, 2010) and a maximum likelihood tree was inferred using IQ-TREE (-m GTR + I + G4) with standard bootstraps (-b 100) (Nguyen et al., 2014).

### Card-Fish

Water samples were pre-filtered through a 20-μm mesh plankton net and fixed in a 2% final concentration of formaldehyde (freshly prepared by filtering through 0.2 μm syringe filter) for at least 3–4 h before filtration. 50 ml of epilimnion and 100 ml of hypolimnion samples were filtered through polycarbonate filters (pore size 0.8 μm, diameter 25 mm, Advantec), rinsed twice with 1X PBS and twice with MilliQ water, air dried and frozen at −20°C until further processing.

CARD-FISH was performed according to the method described in Mukherjee et al. (2015). The filters were embedded in 0.1% low-gelling-point agarose and cut into eight sections, which were hybridized at 35°C for 12 h with a 0.5 μg ml^–1^ probe concentration and a 30% concentration of formamide. The probes (Supplementary Table S1) were purchased from Thermo Electron Co. (Ulm, Germany). Counting was performed using an Olympus BX50 epifluorescence microscope under 1000 × magnification at blue/UV excitation. For the kinetoplastids, either 100 microscopic fields were counted, or when the densities were low the complete filter piece was screened per sample. The total eukaryotes were counted simultaneously with the kinetoplastid cells by DAPI staining under UV excitation.

### Statistical Analysis

Coverage-based rarefaction (Chao and Jost, 2012) was conducted before diversity analysis, where the reads were discarded from each sample until the coverage was 97%, or slope of the rarefaction curve was >0.03, which was the minimum value recorded among the samples. To compare the kinetoplastid communities in the studied lakes, cluster analysis was computed on the rarefied data on a Bray-Curtis similarity matrix using the ‘Vegan’ R package. Subsequently, kinetoplastid diversity in different samples was compared with a diversity index analysis (Shannon) using the ‘Vegan R package’ (Oksanen et al., 2013). Venn diagram was plotted to compare the OTUs of Lake Biwa samples using the ‘Venn diagram’ package for R. All the analyses were computed in R environment^4^ (R Development Core Team, 2013). The distance between kinetoplastid communities in the studied lakes were estimated by an unconstrained ordination analysis, Detrended Correspondence Analysis (DCA) using Canoco v5.0 program package (Ter Braak and Smilauer, 2012) on the rarefied data.

## Results

### Physico-Chemical Parameters

The water columns from all the studied lakes were thermally stratified with the thermocline located at 20–30 m (Figure 2A). The epilimnion temperature of Lakes Biwa and Ikeda were relatively higher at 27°C, whereas the temperatures in the other lakes were 23°, 19° and 18°C in Lakes Chuzenji, Motosu and Sai, respectively. The hypolimnion of Lake Ikeda had the highest temperature of 11°C, followed by 8°, 6°, 5° and 4°C in Lakes Biwa, Motosu, Sai and Chuzenji, respectively. All the lakes had oxygenated hypolimnion except for Lake Ikeda, which has an anoxic hypolimnion near the bottom waters (ca. 200 m). Therefore, hypolimnion samples from Lake Ikeda were taken from the oxygenated layers (50 and 70 m).

### Abundance of Nanoeukaryotes and Kinetoplastids

A high abundance of nanoeukaryotes was found in the epilimnion of all the lakes, where the highest abundance of 9.1 × 10^3^ cells ml^–1^ was found at 5 m in Lake Biwa (Figure 2B). The abundance of nanoeukaryotes reduced with the increase in depth and the lowest abundance was found at 100 m in Lake Motosu (0.5 × 10^3^ cells ml^–1^). In the epilimnion, kinetoplastid flagellates were detected only from Lake Biwa and Lake Ikeda (Figure 2C). The abundance of kinetoplastids was 1.7 × 10^3^ cells ml^–1^ and 1.3 × 10^3^ cells ml^–1^ and contributed up to 18 and 20% of total nanoeukaryotes at 5 and 10 m, respectively in Lake Biwa, and the abundance in Lake Ikeda was 2.9 × 10^2^ cells ml^–1^, and contributed 6% of total nanoeukaryotes (Figure 2C). The contribution of kinetoplastids increased with the increase in depth in all lakes, and they were the dominant members of total nanoeukaryotes in the hypolimnion. The highest abundance of kinetoplastids was detected from the hypolimnion of Lake Biwa with the maximum abundance of 9.3 × 10^2^ cells ml^–1^ at 70 m, contributing up to 54% of total nanoeukaryotes. Similarly, the abundance of kinetoplastids in the hypolimnion of Lake Ikeda was 2.3 × 10^2^ cells ml^–1^ at 50 m and 2.0 × 10^2^ cells ml^–1^ at 70 m, contributing up to 32 and 36% of total nanoeukaryotes, respectively. The maximum abundance of kinetoplastids in the hypolimnion of other lakes was 2.4 × 10^2^ cells ml^–1^ (31% of total nanoeukaryotes) in Lake Chuzenji, 1.5 × 10^2^ cells ml^–1^ (18% of total nanoeukaryotes) in Lake Sai and 9.1 × 10^1^ cells ml^–1^ (19% of total nanoeukaryotes) in Lake Motosu.

### Diversity of Kinetoplastid and Other Euglenozoan Flagellates

**FIGURE 3:**
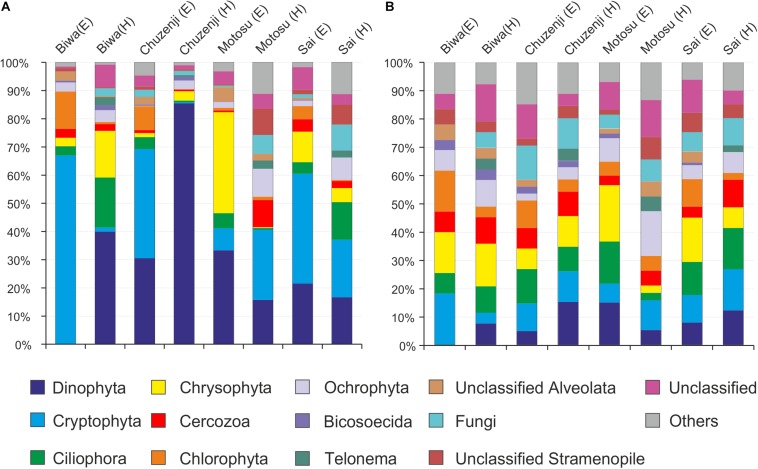
Diversity of nanoeukaryotes in Japanese deep freshwater lakes using universal primers, **(A)** sequence abundance of each OTU and **(B)** OTU abundance (number of OTUs).

**FIGURE 4:**
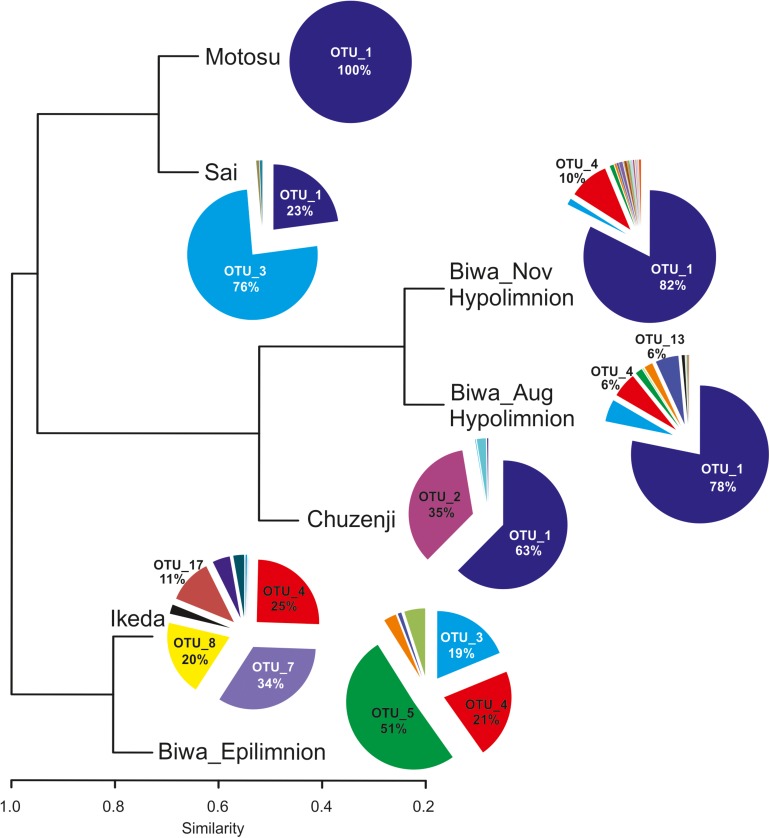
Diversity of kinetoplastids and other euglenozoan flagellates in Japanese deep freshwater lakes using kinetoplastid-specific primers (based on sequence abundance of each OTU). The cluster dendrogram is based on a Bray-Curtis similarity matrix.

A total of 9610 raw reads were obtained with the universal eukaryote primers, from which 7946 sequences remained after quality filtration. Various group of microbial eukaryotes were detected using the universal primers, where Cryptophyta and Dinophyta dominated the epilimnion and hypolimnion communities, respectively, in all the lakes based on the sequence abundance (Figure 3A). However, the major groups were evenly distributed in each lake based on the number of OTUs observed in each group (Figure 3B). Several OTUs specific to epilimnion and hypolimnion with a significant contribution were also detected. Although they had a high abundance, no sequences affiliated to kinetoplastids were detected from any lakes using the universal primers. However, kinetoplastids were readily detected from all the lakes using kinetoplastid-specific primers (Figure 4). Seven samples with kinetoplastid-specific primers produced 9017 raw reads, which yielded 7449 pyrotags after quality filtering. A total of 58 OTUs were obtained, where 17 OTUs were unique to each environment (Table 2). OTUs affiliated to eukaryotes other than the Euglenozoans, were not considered for further analysis. In the Shannon diversity index the highest diversity was observed in the epilimnion of Lake Biwa, followed by Lake Ikeda (Table 2). However, the richness (S.chao1) was the highest in the hypolimnion of Lake Biwa followed by the epilimnion of Lake Biwa (Table 2).

**FIGURE 5:**
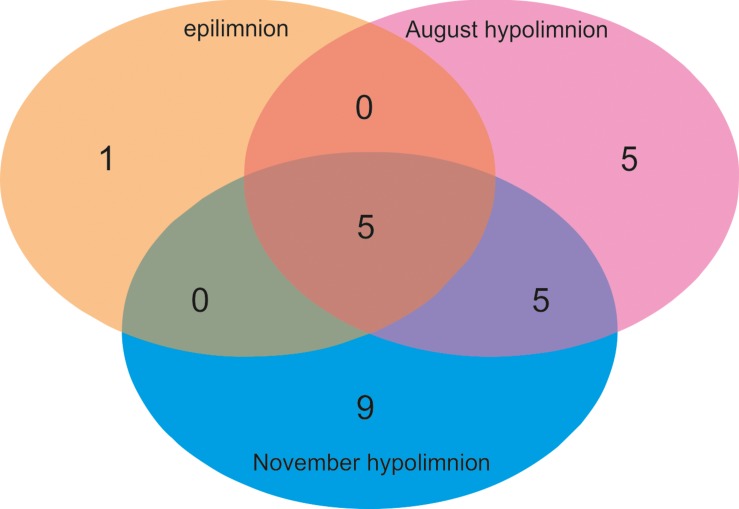
Venn diagram showing the OTU sharing of kinetoplastid flagellates among the epilimnion and hypolimnion of Lake Biwa.

Six OTUs of kinetoplastids were obtained from the epilimnion sample, where the dominant OTU (OTU_5) was closely related to *Rhynchomonas nasuta* (Figure 4 and Supplementary Table S2). Interestingly, the second dominant OTU was affiliated to *Diplonema* sp. (OTU_4), followed by OTU_3, which was closely related to *Azumiobodo hoyamushi*. OTU_15, which was closely related to *Neobodo designis* was only detected from the epilimnion of Lake Biwa, though it was not among the dominant representatives. Two samples were analyzed from the hypolimnion of Lake Biwa to understand the community of the dominant kinetoplastids. Both the samples representing 60 m from August and November showed high number of OTUs with 15 and 19 OTUs, respectively (Table 2). The kinetoplastid community in both the samples was similar with the dominant OTU (OTU_1) being closely related to *Bodo saltans* (Figure 4 and Supplementary Table S2). The OTU affiliated to *Diplonema* sp. (OTU_4), which had a significant contribution in the epilimnion was also dominant in the hypolimnion community. Five out of the six epilimnion kinetoplastid OTUs were present in both the hypolimnion samples (Figure 5). Compared with the epilimnion OTUs, the hypolimnion showed a high number of unique OTUs with 3 in August and 10 in November. The dominant hypolimnion OTU (OTU_1), which contributed 78% and 82% of total sequences in August and November, was not detected from the epilimnion sample.

**FIGURE 6:**
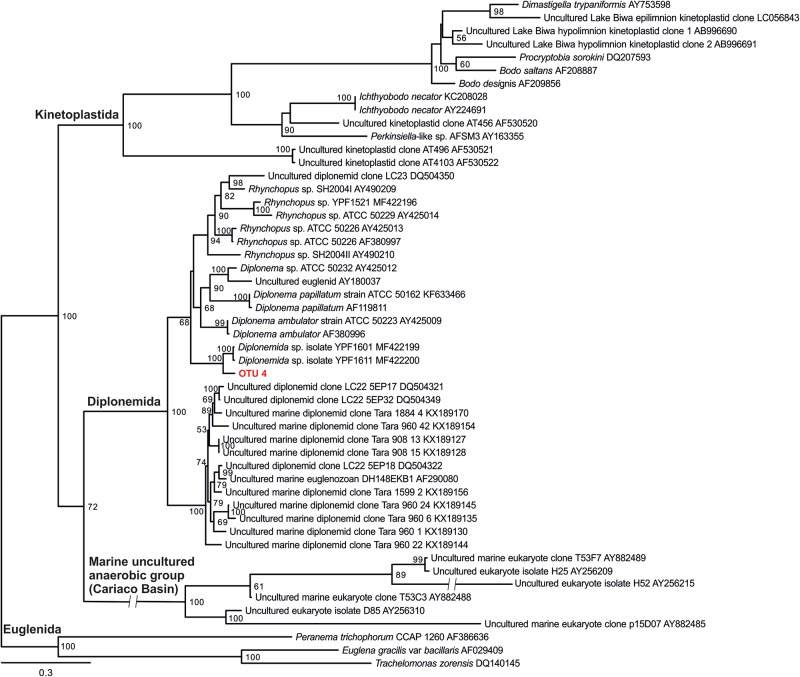
18s rRNA gene phylogenetic tree of diplonemid from freshwater deep lakes of Japan with 28 sequences of cultured and environmental sequences of marine diplonemids. The diplonemid OTU obtained from the present study (OTU 4) is highlighted in bold and red color. Kinetoplastids and other euglenozoan sequences were considered as outgroup. ML tree was inferred with standard bootstraps (-b 100). Scale bar = 0.3 substitution per site.

OTU_1 was also predominant in the hypolimnion of Lakes Chuzenji, Motosu and Sai (Figure 4), contributing 63%, 100% and 23% of total sequence abundance, respectively. However, this OTU was not detected in the hypolimnion of Lake Ikeda. The dominant OTU in the hypolimnion of Lake Ikeda (OTU_7) was closely related to *B. saltans*, contributing 34% of total sequence abundance. The OTU closely related to *Diplonema* sp., that was found in the epilimnion and hypolimnion of Lake Biwa was also detected in the hypolimnion of Lake Ikeda (OTU_4) with a significantly high contribution (25% of total sequence abundance). In phylogenetic analysis, the diplonemid OTU was separated from the marine clones and majority of the cultured diplonemids. However, this OTU was closely related to two diplonemid isolates from the sea around Japan (Figure 6). The Lake Biwa epilimnion kinetoplastid community had the closest similarity with the hypolimnion community of Lake Ikeda (Figure 4), though the similarity was low and the distance was high (Supplementary Figure S2). In contrast, the kinetoplastid community of the Lake Biwa hypolimnion showed a high similarity to the hypolimnion community of Lakes Chuzenji and Motosu (Figure 4 and Supplementary Figure S2).

### Presence of Ubiquitous and Unique OTUs in Each Lake

The most ubiquitous OTU in the present study was OTU_3, which was closely related to *Azumiobodo hoyamushi* and which was detected in all the samples, except for those from Lake Motosu (Figure 4 and Supplementary Table S2). Another ubiquitous OTU (OTU_1), which was closely related to *Bodo saltans* was found in the hypolimnion of all the lakes, except for Lake Ikeda. Three out of the five lakes showed the presence of unique OTUs confined to each environment (Table 2), where the hypolimnion of Lake Biwa had the highest number of unique OTUs (Table 2). The exceptions were Lakes Sai and Motosu, which were inhabited by four and one kinetoplastid OTUs, respectively. The OTUs unique to Lake Biwa (the major ones- OTU_ 5, 10, 13 and 15), Lake Chuzenji (OTU_2) and Lake Ikeda (OTU_7 and 17) had significant contributions to the total kinetoplastid community (Supplementary Table S2). Moreover, several of these unique OTUs and also OTUs obtained from the hypolimnion of all the lakes had less similarity with the closest sequences in the database (Supplementary Table S2).

### Oligotyping of the Dominant OTU

**FIGURE 7:**
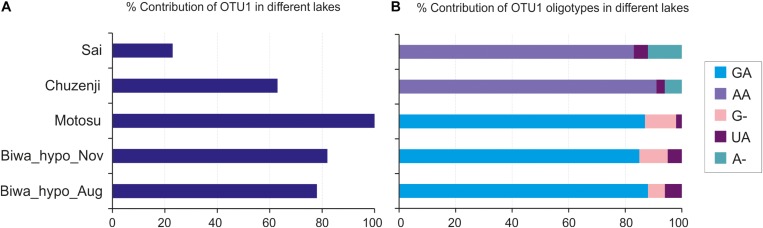
Oligotyping of the dominant kinetoplastid OTU (OTU_1). **(A)** Percentage contribution of OTU_1 in the hypolimnion of studied lakes; **(B)** Percentage contribution of OTU_1 oligotypes. GA, AA, G-, UA and A- represent the detected oligotypes.

Oligotyping of OTU_1, which was dominant in the hypolimnion of four of the five studied lakes (Figure 7A), revealed five oligotypes (GA, G-, UA, AA and A-) (Figure 7B and Supplementary Figure S3). Each lake consisted of three oligotypes, and the oligotype UA was found in all lakes. Lakes Biwa and Motosu shared the same oligotypes, with oligotype GA the dominant representative. Similarly, oligotypes of Lakes Chuzenji and Sai were the same with oligotype AA being the dominant representative.

## Discussion

The abundance of nanoeukaryotes in the studied lakes (Figure 2B) corroborates the results from studies of other deep lakes of the world (Lepère et al., 2010). However, the present study is the first to analyze the abundance and diversity of kinetoplastids in deep freshwater lakes to understand their importance in the hypolimnion waters, which will lead to understanding their ecological role in the freshwater food web. The results of the present study along with the previous studies show that kinetoplastids are important eukaryotes in the hypolimnion of freshwater lakes (Steinberg et al., 1983; Mukherjee et al., 2015), suggesting their importance in matter cycling in deep waters. The dominance of kinetoplastids in the oxygenated hypolimnion of Japanese deep lakes (Figure 2C) and kinetoplastid-like flagellates in some European deep lakes (Steinberg et al., 1983) indicate the possible dominance of this previously underestimated group in the deep freshwater lakes of the world.

### Distribution of Kinetoplastid and Other Euglenozoan Flagellates in Deep Freshwater Lakes

The diversity of a particular group of flagellates is poorly understood and the present study is the first to understand the detailed diversity of a flagellate group using amplicon sequencing. The kinetoplastid communities in the hypolimnion were similar among all the lakes, except for Lake Ikeda (Figure 4 and Supplementary Figure S2), probably due to the relatively warmer water in its hypolimnion (Figure 2A). The communities of the studied lakes were characterized by the presence of both ubiquitous and unique lake-specific OTUs (Figure 4 and Table 2). Identical OTUs of eukaryotic microorganisms were reported from geographically distant sampling stations in oceans (Atkins et al., 2000; Massana et al., 2004). Moreover, dominant and identical kinetoplastid OTUs were also reported from the deep waters of geographically distant parts of the Atlantic Ocean as oceanic waters are interconnected and allow extensive gene flow (Salani et al., 2012) and also owing to the high dispersal potential of the microorganisms, which can overcome geographical and environmental barriers (Finlay, 2002). Even though freshwater lakes are isolated environments, the same genotype of flagellates was reported and isolated from freshwaters of geographically distant countries (Boenigk et al., 2005). Therefore, the presence of ubiquitous OTUs, with some dominant, in the hypolimnion of the studied lakes suggests that dispersal also play a major role in shaping the community of geographically distant freshwater lakes that have similar physico-chemical characteristics.

Detection of unique lake-specific OTUs (Figure 4 and Table 2) was an interesting finding, as these OTUs were not minor taxa and had significant contribution to the kinetoplastid community. However, deeper sequencing and temporal data collection are necessary to conclude about their lake-specificity. Nevertheless, low similarity of these OTUs along with the other hypolimnion OTUs with the related sequences in the database (Supplementary Table S2) indicates that these taxa are potentially new. Near full-length sequences of the dominant hypolimnion kinetoplastids obtained from the present study using clone library analysis and Sanger sequencing, also showed low similarity with the closely related sequences in the database (data not shown).

Detection of one OTU of diplonemids from both Lake Biwa and Ikeda using kinetoplastid-specific primers reveal the close similarity between the two sister groups (Lukeš et al., 2005). No diplonemid OTU was detected using the universal eukaryote primers (Figure 3), indicating that the commonly used universal primers also underestimate diplonemids. Diplonemids are considered as marine flagellates (Lukeš et al., 2015; Tashyreva et al., 2018) and are especially associated with sediments (López-García et al., 2007). Recently, this group has attracted significant interest due to their high abundance and diversity in global oceans, especially in the deep waters (Lara et al., 2009; de Vargas et al., 2015; Flegontova et al., 2016). To the best of our knowledge, diplonemids are not reported from freshwaters and this is the first report on their presence in freshwater lakes. The diplonemid OTU of the present study has less similarity with the marine diplonemids, indicating that freshwater diplonemids are new (Figure 6). However, the relatively close similarity with two marine diplonemid isolates from sea around Japan indicates some evolutionary link between the freshwater and marine diplonemids, which are geographically closely distributed. The detection of diplonemids with a significant contribution in the freshwater lakes and their un-detection using universal eukaryote primers indicates their hidden distribution in freshwater deep lakes.

### Dynamic Community Composition of Kinetoplastid Flagellates in Lake Biwa

Kinetoplastids were found to be dominant in the hypolimnion of Lake Biwa throughout the stratified period (Mukherjee et al., 2015). Although, the overall community in the two hypolimnion samples of Lake Biwa was similar (Figure 4), high OTU richness (Table 2) and the presence of several distinct OTUs showed a variation in the community composition among the less represented OTUs (Figures 4, 5). A study from Lake Fuschlsee has also observed highly variable protist community compositions in the epilimnion, primarily due to the variations in the less represented taxa (Nolte et al., 2010). Flagellate communities in the hypolimnion are known to fluctuate based on the season (Mukherjee et al., 2017), and the fluctuations in the present study were also found within a single group in the stable hypolimnion waters. This indicates that due to the seasonal variation in the hypolimnion communities, the kinetoplastid communities in the studied lakes may also change, especially among the less represented members. Thus, seasonal changes in the individual communities must be studied to understand the community dynamics in more detail.

### Intra-Diversity Within the Dominant OTU

Oligotyping has been conducted in environmental prokaryotic communities to understand the sequence variation within particular OTUs among different environments (Eren et al., 2013; Magnabosco et al., 2014; Newton and McLellan, 2015; Kleindienst et al., 2016; Okazaki et al., 2017). This is the first study in microbial eukaryotes to conduct oligotyping to reveal the intra-diversity within an OTU and their distribution in different lakes. We detected five oligotypes of the dominant hypolimnion OTU with shared and restricted representatives in particular lakes (Figure 7) indicating the role of both dispersal and local environmental conditions in sequence variation in deep waters. However, the distribution pattern of the oligotypes among the lakes hints at the possibility of environmental selection or geographic isolation in playing the major role in structuring the community of microbial eukaryotes, as the shared oligotype was not dominant and the dominant oligotypes were not shared among all the lakes. However, the reason behind the presence of similar oligotypes between Lakes Biwa-Motosu and Chuzenji-Sai is not clear. Nevertheless, intra-diversity within OTUs and their distribution pattern shows interesting information about the adaptation of oligotypes in different lakes, which are otherwise missed when only OTUs are considered. This pattern was well represented in freshwater bacterioplankton communities where oligotypes of ubiquitous bacterial lineages specific to each environment were present in eutrophic and oligotrophic waters (Newton and McLellan, 2015). Thus, oligotyping is an essential tool and more studies on microbial eukaryotes are needed to understand the intra-diversity within the OTUs of ubiquitous lineages from different trophic levels or from isolated environments or environments separated by geographic distance. In the present study oligotyping was conducted at OTU levels, separated at 97% cut-off. Although the cut-off was 97%, the similarity among the sequences in OTU1 (OTU used for oligotyping in the present study) was still more than 99% (the commonly used OTU level cutoff in microbial eukaryotic diversity studies).

### Undetection of Euglenozoan Flagellates in Diversity Studies Using Universal Primers

Kinetoplastids were overlooked in clone libraries by commonly used universal eukaryote primers (Mukherjee et al., 2015), and in the present study these flagellates were also overlooked using amplicon sequencing with another commonly used universal eukaryote primer pair (Stoeck et al., 2010) (Figure 3). Therefore, the frequently used universal primers employed to study the diversity of microbial eukaryotes (Massana et al., 2015; Pernice et al., 2016) overlook an important group, irrespective of the sequencing platform used. Moreover, undetection of diplonemids in the present study by the universal primers indicate the underestimation of Euglenozoans in diversity studies worldwide due to their highly divergent 18S rRNA gene (Simpson et al., 2006; Bochdanksy and Huang, 2010; Mukherjee et al., 2015). A recent study has reported that similar to kinetoplastids, diplonemids also have mismatches in the conserved region of the 18S rRNA gene (Bochdansky et al., 2017). This indicates that the contributions of several important groups in the ecosystem are overlooked owing to their underestimation by the universal primers. One of the limitations of the present study is that we could not collect seasonal samples from each lake to be able to understand the fluctuations in the community in detail. Thus, seasonal studies in particular groups should be conducted to understand the dynamics and importance of individual groups.

## Conclusion

The use of 18S amplicon sequencing in the present study helped to unravel the detailed diversity of a dominant group of flagellates in various deep freshwater lakes. High diversity of a particular group and presence of oligotypes within an OTU suggest that flagellate communities are complex and more studies are required with a focus on individual groups. The results of the present study suggest the need for further studies to understand the probable dominance of these cosmopolitan flagellates in the hypolimnion of other deep lakes of the world. The complete ignorance of kinetoplastid flagellates with universal primers even with high amplicon sequencing indicates the global ignorance of an important group. Moreover, detection of diplonemids from freshwater lakes shows their presence in freshwaters and indicates the underestimation of euglenozoan flagellates in molecular diversity studies. Studies on particular groups are necessary due to the limitation of universal primers in being able to target phylogenetically diverse groups and also to understand the importance of individual groups to better understand the ecosystem processes.

## Data Availability Statement

The datasets generated for this study can be found in the DDBJ Sequence Read Archive (DRA) database, Bio-project accession number: PRJDB6819.

## Author Contributions

IM, YH, and S-iN contributed to conception and design of the study. IM, YH, SF, and KO conducted the sampling. IM conducted the experiments. YH and KO helped in some analysis. IM, YO, and SF processed and analyzed the data. IM drafted the manuscript. All authors contributed to critical revisions and approved the final version of the manuscript.

## Conflict of Interest

The authors declare that the research was conducted in the absence of any commercial or financial relationships that could be construed as a potential conflict of interest.

## Footnotes

1 www.arb-silva.de/

2 https://blast.ncbi.nlm.nih.gov/Blast.cgi?PROGRAM=blastn&PAGE_TYPE=BlastSearch&LINK_LOC=blasthome

3 http://merenlab.org/software/oligotyping/

4 www.r-project.org
