# Supplementary material for: Widespread Dominance of Kinetoplastids and Unexpected Presence of Diplonemids in Deep Freshwater Lakes
Source: Front Microbiol. 2019 Oct 16;10:2375. doi: 10.3389/fmicb.2019.02375 (PMC6805782; doi:10.3389/fmicb.2019.02375)
Supplement: Supplementary file 1 [file Table_1.pdf]

Supplementary Material

1 Supplementary figures

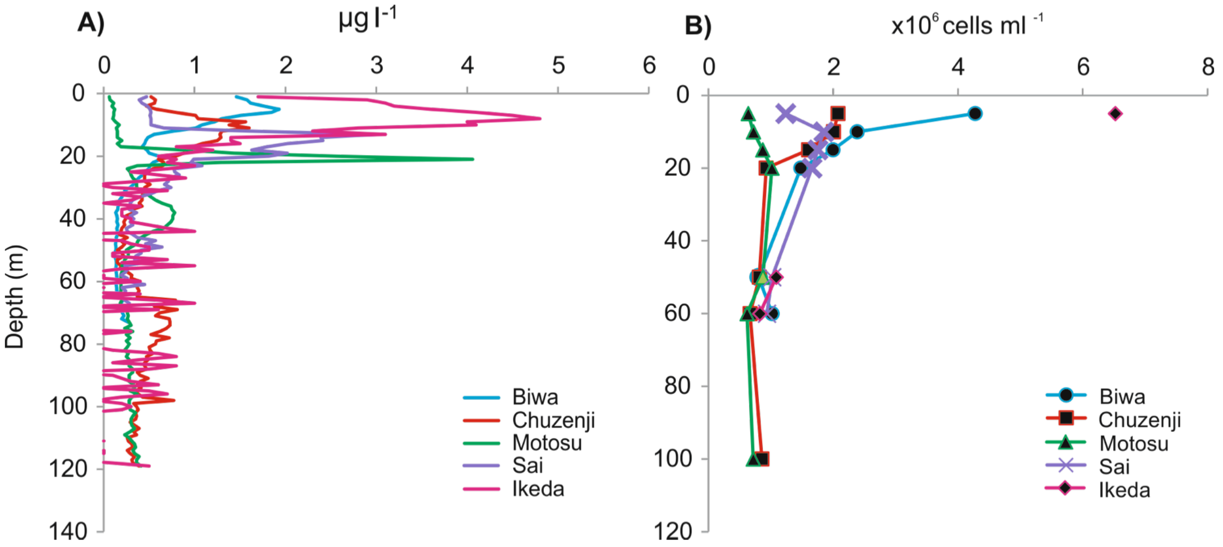

1.1 Supplementary Figure S1: Vertical distribution of (A) concentration of chlorophyll *a* and (B) abundance of bacteria in the studied lakes.

Concentration of chlorophyll *a*

Concentration of chlorophyll *a* varied in each lake with the chlorophyll maxima around 10-20 m (Figure S1A). The highest concentration chlorophyll *a* was found in Lake Ikeda (4.8 µg l^{-1}) followed by Lake Motosu (4.1 µg l^{-1}), Sai (3.0 µg l^{-1}), Biwa (2.0 µg l^{-1}), and Chuzenji (1.6 µg l^{-1}). Concentration of chlorophyll *a* decreased with the depth, with the lowest values observed in the deepest layers.

Abundance of bacteria

Bacterial abundance was high in the epilimnion of all the lakes and reduced with the increase in depth (Figure S1B). The highest abundance was found at 5 m of Lake Ikeda with 6.5×10^6 cells ml^{-1}. The highest abundance in each lake was 4.3×10^6 cells ml^{-1} at 5 m of Lake Biwa, 2.1×10^6 cells ml^{-1} at 5 m of Lake Chuzenji, 1.9×10^6 cells ml^{-1} at 10 m of Lake Sai, 1.0×10^6 cells ml^{-1} at 20 m of Lake Motosu. The abundance of bacteria decreased with the increase in depths (below the thermocline) in all the lakes, where the average abundance in the hypolimnion was 1.5×10^6 cells ml^{-1} in Biwa, 1.0×10^6 cells ml^{-1} in Ikeda, 1.0×10^6 cells ml^{-1} in Sai, 0.8×10^6 cells ml^{-1} in Chuzenji, and 0.7×10^6 cells ml^{-1} in Motosu.

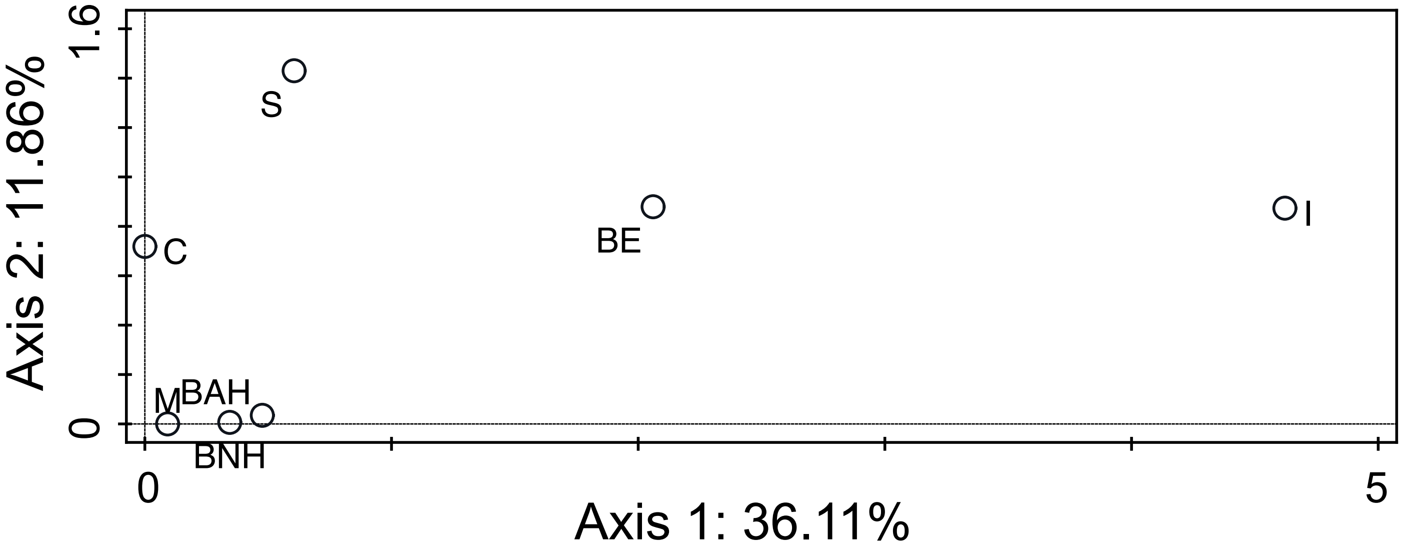

1.2 Supplementary Figure S2: Ordination plot of Detrended Correspondence Analysis (DCA) explaining the distance between the kinetoplastid communities in the studied lakes. The proportions of the total variability explained by first two axes are given. BE: Biwa epilimnion, BAH: Biwa August hypolimnion, BNH: Biwa November hypolimnion, M: Motosu, C: Chuzenji, S: Sai, I: Ikeda.

>GA

CUAAG-CCAUGCAUGCCUCAGAA-UCACUGCAUU-GCAGGA-AUCUGCGCAUGG-
CUCAUU-ACAUC-A--G-ACGU-AA--UCUGCCG-CAAAAAU-UUGCGGUUUCCGCA-
UAUUGGAUACC-UUGG-CGAAA-C-GCCAA-G-CUAA-UACA-U-GAA-CAAACAGGAU--
AUC-CUCC-GA--UAAACAGUGUG-A-GGUUC--GCCGAGC-----AU-UGUUGCGA--GGU-G--
U-CCAGCG-AA-U-GAAU-GAAAU--UAA-AACC-AAUGCCGCGG---ACGCG-GCAGCA-
GCACC-CAG----AAG-UGUU-G-A-CCCAAUUC-AUUCCGUG-CGAAAG-CCG-G-UUU-UC-
CGGCGU-C--UUU-UG

>AA

CUAAG-CCAUGCAUGCCUCAGAA-UCACUGCAUU-GCAGGA-AUCUGCGCAUGG-
CUCAUU-ACAUC-A--G-ACGU-AA--UCUGCCG-CAAAAAU-UUGCGGUUUCCGCA-
UAUUGGAUACC-UUGG-CGAAA-C-GCCAA-G-CUAA-UACA-U-GAA-CAAACAGGAU--
AUC-CUCC-GA--UAAACAGUGUA-A-GGUUC--GCCGAGC-----AU-UGUUGCGA--GGU-G--
U-CCAGCG-AA-U-GAAU-GAAAU--UAA-AACC-AAUGCCGCGG---ACGCG-GCAGCA-
GCACC-CAG----AAG-UGUU-G-A-CCCAAUUC-AUUCCGUG-CGAAAG-CCG-G-UUU-UC-
CGGCGU-C--UUU-UG

>G-

CUAAG-CCAUGCAUGCCUCAGAA-UCACUGCAUU-GCAGGA-AUCUGCGCAUGG-
CUCAUU-ACAUC-A--G-ACGU-AA--UCUGCCG-CAAAAAU-UUGCGGUUUCCGCA-
UAUUGGAUACC-UUGG-CGAAA-C-GCCAA-G-CUAA-UACA-U-GAA-CAAACAGGAU--
AUC-CUCC-GA--UAAACAGUGUG-A-GGUUC--GCCGAGC-----AU-UGUUGCGA--GGU-G--
U-CCAGCG-AA-U-GAAU-GAAAU--UAA-A-CC-AAUGCCGCGG---ACGCG-GCAGCA-
GCACC-CAG----AAG-UGUU-G-A-CCCAAUUC-AUUCCGUG-CGAAAG-CCG-G-UUU-UC-
CGGCGU-C--UUU-UG

>UA

CUAAG-CCAUGCAUGCCUCAGAA-UCACUGCAUU-GCAGGA-AUCUGCGCAUGG-
CUCAUU-ACAUC-A--G-ACGU-AA--UCUGCCG-CAAAAAU-UUGCGGUUUCCGCA-
UAUUGGAUACC-UUGG-CGAAA-C-GCCAA-G-CUAA-UACA-U-GAA-CAAACAGGAU--
AUC-CUCC-GA--UAAAC-AAUGU-G-AG-----G-UUC--GCC--GAGCAUUGUUGCGAGGUG--
U-CCAGCG-AA-U-GAAU-GAAAU--UAA-AACC-AAUGCCGCGG---ACGCG-GCAGCA-
GCACC-CAG----AAG-UGUU-G-A-CCCAAUUC-AUUCCGUG-CGAAAG-CCG-G-UUU-UC-
CGGCGU-C--UUU-UG

>A-

CUAAG-CCAUGCAUGCCUCAGAA-UCACUGCAUU-GCAGGA-AUCUGCGCAUGG-
CUCAUU-ACAUC-A--G-ACGU-AA--UCUGCCG-CAAAAAU-UUGCGGUUUCCGCA-
UAUUGGAUACC-UUGG-CGAAA-C-GCCAA-G-CUAA-UACA-U-GAA-CAAACAGGAU--
AUC-CUCC-GA--UAAACAGUGUA-A-GGUUC--GCCGAGC-----AU-UGUUGCGA--GGU-G--
U-CCAGCG-AA-U-GAAU-GAAAU--UAA-A-CC-AAUGCCGCGG---ACGCG-GCAGCA-
GCACC-CAG----AAG-UGUU-G-A-CCCAAUUC-AUUCCGUG-CGAAAG-CCG-G-UUU-UC-
CGGCGU-C--UUU-UG

1.3 Supplementary Figure S3: Sequence of five oligotypes (GA, AA, G-, UA, A-) of the dominant kinetoplastid OTU (OTU_1).

2 Supplementary tables

2.1 Supplementary Table S1: CARD-FISH probes used in the present study

Probes	Sequence (5’–3’) of probes	Position*	Target group	References

KIN516	ACCAGACTTGTCCTCC	502–517	kinetoplastids	Bochdansky and Huang (2010)
EUK516 Competitor	ACCAGACTTGCCCTCC	502–517	–	Bochdansky and Huang (2010)

* positions refer to the 18S rRNA gene of *S. cerevisiae*.

2.2 Supplementary Table S2: Distribution and classification of euglenozoan OTUs obtained in the present study.

Sr. No.	OTU ID	Total sequences	BE	BAH	BNH	C	S	M	I	Closest match	Closest similarity (%)
1	OTU_1	3914	0	1218	854	1594	67	181	0	*Bodo saltans*	90
2	OTU_2	893	0	0	0	893	0	0	0	Bodonidae	91
3	OTU_3	409	86	81	16	3	222	0	1	*Azumiobodo hoyamushi*	87
4	OTU_4	362	98	91	101	0	0	0	72	*Diplonema* sp.	93
5	OTU_5	269	233	26	10	0	0	0	0	*Rhynchomonas nasuta*	89
6	OTU_6	60	0	2	0	58	0	0	0	*Rhynchomonas nasuta*	95
7	OTU_7	96	0	0	0	0	0	0	96	*Bodo saltans*	88
8	OTU_8	58	0	2	0	0	0	0	56	*Rhynchomonas nasuta*	100
9	OTU_10	50	14	31	5	0	0	0	0	*Neobodo designis*	99

10	OTU_13	91	4	84	3	0	0	0	0	*Neobodo borokensis*	99
11	OTU_15	23	23	0	0	0	0	0	0	*Neobodo designis*	84
12	OTU_16	19	0	11	1	0	0	0	7	*Neobodo designis*	85
13	OTU_17	33	0	0	0	0	0	0	33	*Dimastigella trypaniformis*	100
14	OTU_20	20	0	0	0	7	0	0	13	*Rhynchobodo* sp.	95
15	OTU_23	9	0	0	9	0	0	0	0	*Petalomonas cantuscygni*	88
16	OTU_24	6	0	5	1	0	0	0	0	*Rhynchomonas nasuta*	86
17	OTU_26	5	0	2	1	0	2	0	0	*Bodo saltans*	88
18	OTU_28	9	0	1	0	0	0	0	8	*Rhynchomonas nasuta*	98
19	OTU_29	6	0	0	6	0	0	0	0	Uncultured	83
20	OTU_32	3	0	1	2	0	0	0	0	*Crithidia mellificae*	86
21	OTU_33	3	0	0	3	0	0	0	0	Uncultured kinetoplastid	92
22	OTU_34	7	0	0	7	0	0	0	0	Uncultured euglenozoa	98
23	OTU_35	3	0	0	3	0	0	0	0	*Rhynchomonas nasuta*	86
24	OTU_37	2	0	2	0	0	0	0	0	*Neobodo designis*	81
25	OTU_40	2	0	0	2	0	0	0	0	*Neobodo designis*	88

26	OTU_41	3	0	1	0	0	2	0	0	*Rhynchomonas* sp	92
27	OTU_43	2	0	0	2	0	0	0	0	*Neobodo saliens*	87
28	OTU_44	5	0	0	5	0	0	0	0	*Bodo designis*	89
29	OTU_47	5	0	0	5	0	0	0	0	*Neobodo designis*	84

BE: Biwa epilimnion, BAH: Biwa August hypolimnion, BNH: Biwa November hypolimnion, C: Chuzenji, S: Sai, M: Motosu, I: Ikeda. Numbers under each lake represent the total sequences obtained for a particular OTU. Closest match and similarity percentage were obtained from BLAST searches against the NCBI database.
